# Supplementary material for: The association between mineralised tissue formation and the mechanical local in vivo environment: Time-lapsed quantification of a mouse defect healing model
Source: Sci Rep. 2020 Jan 24;10:1100. doi: 10.1038/s41598-020-57461-5 (PMC6981157; doi:10.1038/s41598-020-57461-5)
Supplement: Supplementary file 1 — Supplementary information [file 41598_2020_57461_MOESM1_ESM.pdf]

The association between mineralised tissue formation and the mechanical local *in vivo* environment: Time-lapsed quantification of a mouse defect healing model

Duncan C Tourolle né Betts[1], Esther Wehrle[1], Graeme R Paul[1], Gisela A Kuhn[1], Patrik Christen[1],

Sandra Hofmann[1,2,3], Ralph Müller[1]

[1] Institute for Biomechanics, ETH Zurich, Zurich, Switzerland, [2] Department of Biomedical Engineering and [3] Institute for Complex Molecular Systems, Eindhoven University of Technology, Eindhoven, The Netherlands.

Corresponding author:

Ralph Müller, PhD

Institute for Biomechanics

ETH Zurich

Leopold-Ruzicka-Weg 4

8093 Zurich, Switzerland

Email: ram@ethz.ch

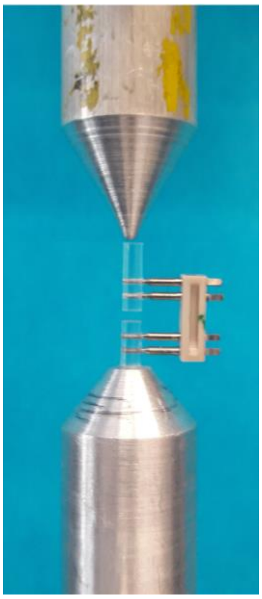

Supplementary Fig. S1. Each fixator was assembled and inserted into a PMMA testing rod. The rod was then placed in a custom designed holder on a ZwickRoell II compression tester (ZwickRoell, Ulm, Germany) and compressed quasi-statically to 1 N (preload of 0.05 N) thrice. A 10 N load cell was used (sensitivity of 0.5 %) and the moving platen tip was rounded to prevent non-axial forces and moments upon the specimen. Reported stiffness is the mean of the gradients of the linear force-displacement curve for the three tests in N/mm.

Supplementary Table S1. The mean measured stiffness of the PMMA-Fixator constructs.

Fixator Number	Fixator Stiffness (N/mm)
1	23.50
2	23.51
3	23.59
4	23.43
5	23.60
6	23.95
7	22.96
8	23.34
9	22.99
10	24.56
11	25.17
12	25.29
Mean	23.82
Standard deviation	0.74
